# Human Hepatitis B Virus Core Protein Inhibits IFNα-Induced IFITM1 Expression by Interacting with BAF200

**DOI:** 10.3390/v11050427

**Published:** 2019-05-09

**Authors:** Tongya Li, Zunlong Ke, Weiyong Liu, Ying Xiong, Ying Zhu, Yingle Liu

**Affiliations:** 1 College of Life Sciences, Wuhan University, Wuhan 430072, China; tongya@whu.edu.cn (T.L.); zke@mrc-lmb.cam.ac.uk (Z.K.); wyliu@hust.edu.cn (W.L.); xiongying@whu.edu.cn (Y.X.); yingzhu@whu.edu.cn (Y.Z.); 2 Structural Studies Division, MRC Laboratory of Molecular Biology, Francis Crick Avenue, Cambridge Biomedical Campus, Cambridge CB2 0QH, UK; 3 Department of Clinical Laboratory, Tongji Medical College, Huazhong University of Science and Technology, Wuhan 430030, China

**Keywords:** Human hepatitis B virus core protein (HBc), BRG1/hBRM-associated factors 200 (BAF200), interferon α (IFNα), interferon-induced transmembrane protein 1 (IFITM1)

## Abstract

Human hepatitis B virus core protein (HBc) is a structural protein of the hepatitis B virus (HBV) and contributes to HBV regulation of host-cell transcription. However, the mechanisms of transcriptional regulation remain poorly characterized. To dissect the function of HBc, a yeast two-hybrid was performed to identify HBc-binding proteins, and the C-terminal of BRG1/hBRM-associated factors 200 (BAF200C) was identified. Then, the existence of HBc interactions with BAF200C and full-length BAF200 was confirmed via co-immunoprecipitation assays in 293T, HepG2 and HepG2-NTCP cells. Furthermore, we show that the binding between HBc and BAF200 was of vital importance to HBc mediated downregulation of interferon-induced transmembrane protein 1 (IFITM1) expression, and the mechanisms for the downregulation were disclosed as follows. Basal level of IFITM1 expression depends on BAF200, rather than the JAK–STAT1 pathway. The interaction of HBc with BAF200 disturbs the stability of the polybromo-associated BAF (PBAF) complex and results in the suppression of IFTM1 transcription. Finally, the antiviral effects of IFITM1 on cell proliferation and HBV replication were found to be partially restored when HBc was co-transfected with BAF200. Collectively, our findings indicate that HBc plays a role in HBV resistance against the antiviral activities of IFNα, providing details about HBV evasion of host innate immunity.

## 1. Introduction

The human hepatitis B virus (HBV) is a double stranded DNA virus in the *Hepadnaviridae* family [1]. HBV infection could cause acute and chronic Hepatitis B (CHB), which can progress to cirrhosis and hepatocellular carcinoma, leading to high mortality rates worldwide. Antiviral therapy with interferon aims to induce permanent immune control of HBV infection through stimulation of the hosts’ innate immune response. Nevertheless, experimental data from HBV infected chimpanzees and urokinase-type plasminogen activator/severe combined immunodeficiency (uPA-SCID) mice have shown that HBV infection does not induce an intrahepatic innate immune response that can be detected [2,3]. This is because early in infection it acts like a stealth virus, remaining undetected and spreading until the onset of the adaptive immune response several weeks later [4]. Besides acting as a stealth pathogen, recent developments have shown that HBV can avoid recognition by the host innate immune system. However, the precise mechanisms are largely unknown.

**Figure 1:**
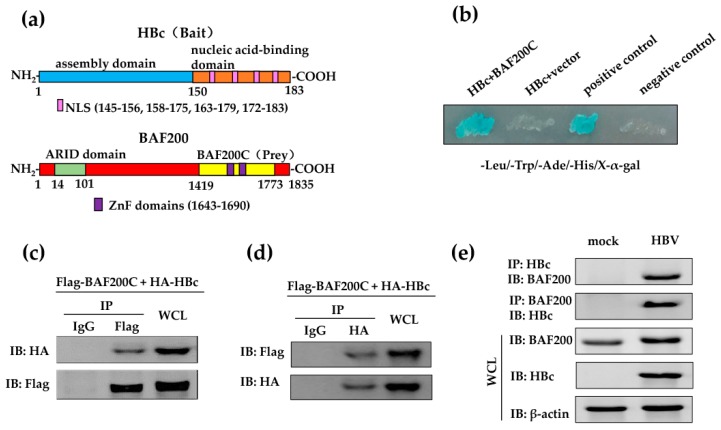
HBc interacts with BAF200. (**a**) Schematic diagram of the bait and prey in the yeast two-hybrid screening. The full length HBc was used as bait for the screening and the C-terminal of BAF200 (BAF200C) was identified as prey. The numbers indicate the location of the amino acids on the BAF200 protein. (**b**) HBc-interacting partners were tested for β-galactosidase activity on an SD/-Leu/-Trp/-Ade/-His/X-α-gal plate. Vector: pGADT7; Positive control: pGBKT7-53 and pGADT7-T co-transformant; Negative control: pGBKT7-lam and pGADT7-T transformant. (**c**) 293T cells were co-transfected with pCMV-Flag-BAF200C and pCMV-HA vector or pCMV-HA-HBc, and co-IP assays were performed with anti-Flag antibody or control IgG. Immuno-complexes were detected by western blot assays using the anti-HA antibody or anti-Flag antibody (control). (**d**) HepG2 cells were co-transfected with the pGC-FU-Flag-BAF200 and pCMV-HA vectors or pCMV-HA-HBc, and co-IP assays were carried out with anti-HA antibody or IgG. Immuno-complexes were detected by western blot assays using the anti-Flag antibody or anti-HA antibody (control). (**e**) HepG2-NTCP cells were incubated with supernatants isolated from the supernatants of HepaAD38 cells cultures (containing HBV) at 1000 GEq for 16 h or not (mock). Co-IP assays were performed with HBc antibody or BAF200 antibody, immuno-complexes were detected by western blot assays using BAF200 antibody or HBc antibody. Input control assays were performed in whole cell lysates (WCL).

Human hepatitis B virus core protein (HBc) is 183 amino acids in length and dimeric in solution [5]. HBc dimers assemble into T = 4 (120 copies) or T = 3 (90 copies) capsids of HBV, with T = 4 capsids being the predominant form in vivo [6]. HBc is composed of an assembly domain (aa 1–149) and a nucleic acid-binding domain (aa 150–183) (Figure 1a), moreover, it not only acts as a structural protein of HBV, but also works as an essential regulator in viral replication [5,7]. The nucleic acid-binding harbors a nuclear localization sequence (NLS), which mediates the transport of HBc into the nucleus [7,8]. In vitro studies have shown that HBc binds directly to the covalently closed circular DNA (cccDNA) upon entering the nucleus, such as the cAMP response element of HBV, Enh I [9], and the nuclear factor kappa B binding site of HBV, Enh II [10], to regulate HBV transcription. In addition, HBc appears to regulate the activities of host cells by interacting directly with the host genome [11]. However, the details of HBc function in host transcriptional regulation are not well understood.

Human SWI/SNF (mating-type switching (SWI) and sucrose non-fermenting (SNF)) complexes regulate the expression of numerous interferon (IFN)-inducible genes by mediating ATP-dependent chromatin remodeling, exposing the binding sites to the transcriptional machinery. SWI/SNF complexes are critical for proliferation, differentiation, tumorigenesis, and DNA repair [12]. There are two forms of SWI/SNF complexes: BRG1/hBRM-associated factors (BAF) and polybromo-associated BAF (PBAF). Only PBAF can facilitate the ligand-dependent transcriptional activation by interacting with the nuclear receptors [13]. BAF and PBAF complexes share most of their subunits and are distinguished by the presence of two specific subunits—BAF180 and BAF200—both of which only exist in PBAF [14]. Furthermore, BAF200, but not BAF180, is essential for the stability of PBAF, and the depletion of BAF200 leads to the complete inactivation of PBAF [13]. BAF200 is encoded by *ARID2*. Besides the N-terminal AT-rich interactive domain (ARID), BAF200 contains multiple LXXLL motifs, which have been shown to participate in the regulation of protein–protein interaction [15]. Additionally, BAF200 has been reported to be a potential tumor suppressor and involved in the IFN signal pathway [16].

IFNs are essential components of the innate immune response and act as the first line of defense against invading microorganisms or pathogens. Interestingly, HBV escapes the host innate immune response merely by preventing the induction of IFNs [17]. IFNs modulate host defenses against microbial infection through the induction of IFN-stimulated genes (ISGs) by the Janus kinase (JAK)–signal transducer and activator of transcription (STAT) signaling pathway. Among these ISGs, interferon-induced transmembrane protein (IFITM) 1, 2, and 3, which are a cluster of genes encoding membrane proteins, exhibit antiviral capabilities mainly through the inhibition of virus entry [18]. IFITM1 restricts the infection of various viruses, including type 1 human immunodeficiency virus [19], hepatitis C virus [20], severe acute respiratory syndrome (SARS) coronavirus [21], and influenza A virus [21,22]. However, whether IFITMs inhibit HBV infection has not been reported. In this study, we start with the discovery that HBc can interact with BAF200. Then, we focus on functions of the interaction and disclose that overexpressed HBc downregulates the BAF200-dependent expression of IFITM1 via disruption of PBAF complex stability. Finally, our data demonstrates that the antiviral effects of IFITM1 on cellular proliferation and HBV replication are partially restored when HBc is co-expressed with BAF200 in HBV-infected cells. These findings enrich details about how HBV counteracts human natural immunity, revealing a potential target for novel therapeutic strategies of HBV infection.

## 2. Materials and Methods

### 2.1. Plasmids

BAF200C (4255–5319 nt of *ARID2*) was amplified from the fragment screened by the yeast two-hybrid system using primers—sense, 5’-GATCCATGGCAAACTCGACGGGGAA-3’ and antisense, 5’-AATTCTCACTGCAGCATTTCTGA-3’—and inserted into a pCMV-Flag vector (Stratagene, San Diego, CA, USA). BAF200 (full-length *ARID2*) and HBc cDNAs (Taxonomy ID: 489463) were synthesized by GenScript Co. Ltd. (Nanjing, China). pGC-FU-Flag and the pHBV1.31 vector were kindly gifted by Prof. R Xiang (Xiangya School of Medicine of Central South University, China). The pGC-FU-Flag vector was ligated and constructed with BAF200 at restriction enzyme site *Age*I. The full-length of HBc was cloned into the pGBKT7 (Clontech, Clontech Laboratories, Inc., Mountain View, CA, USA) vector with primers—sense, 5’-ACTTCCAGACTTCTAGGGAGAC-3’ and antisense, 5’-CTGCCCTGTGACGGAATTGA-3’—and cloned into the pCMV-HA vector (Clontech) with primers—sense, 5’-GATCCATGGACATTGACCACTATAAA-3’ and antisense, 5’-TCGACCTAACATTGAGATTCCCGAGA-3’.

### 2.2. Yeast Two-Hybrid Assay

The Matchmaker GAL4 Two-Hybrid System 3 (Clontech) was used for the screening of a human fetal brain cDNA library (Clontech) with pGBKT7-HBc as bait. Co-transformants were selected on synthetic dropout (SD), media lacking leucine, and tryptophan (SD/-Leu/-Trp) and were validated by growth on SD, media lacking leucine, tryptophan, adenine, and histidine (SD/-Leu/-Trp/-Ade/-His) and containing 5-bromo-4-chloro-3-indolyl-α-D-galactoside (X-α-gal). Then, positive colonies were sequenced (Invitrogen, Carlsbad, CA, USA).

### 2.3. Cells Culture and Transfection

HepG2 cells and 293T cells, purchased from CCTCC, were cultured in Dulbecco’s modified Eagle medium (DMEM, Gibco, Thermo Fisher Scientific, Waltham, MA, USA), supplemented with 10% (*v*/*v*) fetal bovine serum ((FBS, Gibco), 1% penicillin and 0.1 mg/mL streptomycin in a humidified incubator maintained at 37 °C with 5% CO_2_. HepG2.2.15 cells, obtained from Prof. R Xiang, were cultured in 1640 medium (Gbico) in the presence of G418 (200 μg/mL, Sigma-Aldrich, St. Louis, MO, USA) in a humidified incubator maintained at 37 °C with 5% CO_2_. HepG2-NTCP cells and HepaAD38 cells, provided by Prof. Y Zhu (Wuhan University, China), were cultured in DMEM (Gibco), supplemented with 10% heat-inactivated fetal calf serum (Gibco), 100 U/mL penicillin, and 100 μg/mL streptomycin sulfate at 37 °C in 5% CO_2_. All the transfection reactions were performed on indicated cells (2 × 10^6^) in log phase, using Lipofectamine 2000 (Invitrogen) according to the manufacturer’s protocol. Co-transfection was performed using a total of 5 µg of plasmids or vectors in a 1:1 (*w*/*w*) ratio: the pCMV-HA-HBc or pCMV-HA vectors were co-transfected with pCMV-Flag-BAF200C or pGC-FU-Flag-BAF200 into indicated cells. The medium was refreshed with serum-free DMEM/1640 6 h after transfection. The supernatant was harvested 36 h post incubation for co-immunoprecipitation or western blot analyses.

### 2.4. Viruses and Infection

The supernatants of HepaAD38 cells were concentrated 100-fold by ultracentrifugation as HBV inoculums. HBV stock titer (genome equivalents [GEq] per milliliter) was measured by using qPCR.

For infection, HepG2-NTCP cells of a low passage number were seeded onto precooling collagen I-coated plates and incubated in DMEM for 6 h, then the medium was replaced by primary hepatocyte maintenance medium (PMM) with 2% FBS (Gibco) for 12 h. After this, cells were infected with 1000 GEq per cell of HBV in PMM containing 4% (*w*/*v*) polyethylene glycol 8000 (PEG 8000) for 16 h. After the virus-containing medium was removed, cells were washed several times and cultured in fresh PMM. The medium was changed every other day [23,24]. PMM is Williams’ E medium supplemented with an insulin-transferrin-selenium solution (Thermo, Waltham, MA, USA), 10 ng/mL of human epidermal growth factor (EGF, Peprotech, Rocky Hill, NJ, USA), 2 mM l-glutamine (Thermo), 18 μg/mL of hydrocortisone, 2% dimethyl sulfoxide (DMSO), 40 ng/mL of dexamethasone, 100 μg/mL of streptomycin, and 100 U/mL of penicillin.

### 2.5. Co-Immunoprecipitation Assay

After transfection, cells were washed with ice-cold PBS after 36 h, harvested by scraping, then lysed using an RIPA lysis buffer (50 mM Tris-HCl [pH 7.4], 150 mM NaCl, 1% NP40, 0.1% SDS) supplemented with a protease inhibitor cocktail (Thermo Scientific). After centrifugation at 13,000 rpm at 4 °C for 5 min, supernatants were incubated with primary antibody or IgG (Santa Cruz, Santa Cruz, CA, USA) and protein G agarose beads (GE Healthcare, Chicago, IL, USA) at 4 °C overnight. Immunoprecipitates were washed with a washing buffer (50 mM Tris-HCl [pH 7.4], 150 mM NaCl) three times, then whole cell lysate and immunoprecipitated fractions were used for western blot analysis. The following primary antibodies were used: anti-HA (catalog no. H6908, Sigma-Aldrich), anti-Flag (catalog no. F1804, Sigma-Aldrich), anti-BAF200 (catalog no. A302-230A, Bethyl), anti-HBc (catalog no. B0586, Dako).

### 2.6. Western Blot

In order to make sure the loading amounts of the protein were comparable, the protein concentration was quantified by Bradford assay [25]. Thirty micrograms of protein was separated by SDS polyacrylamide gel electrophoresis and then transferred to polyvinylidene difluoride (PVDF, Thermo Scientific) membranes. After blocking with 5% gelatin in TBST, the membranes were incubated with the indicated primary antibodies at 4 °C overnight. The membranes were washed three times, incubated with secondary antibodies (1:8000) conjugated with horseradish peroxidase (HRP) for 1 h at room temperature, and visualized with the ECL system (Biorad, Hercules, CA, USA) according to manufacturer’s instructions. Blots were probed with HRP-conjugated secondary antibodies. The following antibodies were used: anti-IFITM1, 2, 3, (catalog no.13126, 13530, 59212, Cell Signaling Technology, Danvers, MA, USA), anti-BAF180 (catalog no. A301-591A, Bethyl), anti-STAT1 (catalog no. 9172, Cell Signaling Technology), and anti-phospho-STAT1 (catalog no. 9167, Cell Signaling Technology), and β-actin (catalog no. 60008-1-Ig, Proteintech Group). The gray density of the western blots was measured by using ImageJ software (National Institutes of Health, Bethesda, MD, USA).

### 2.7. Reverse Transcription Polymerase Chain Reaction (RT-PCR) and Real-Time Quantitative PCR (RT-qPCR)

RT-PCR assays were performed to determine the relative mRNA levels. Total RNA was extracted by TriZol (Invitrogen) according to the manufacturer’s instructions. The quantity of the RNA samples was detected by Nanodrop 2000 (Thermo scientific). One microgram of RNA was reverse transcribed using random hexamer primers (Fermentas, Waltham, MA, USA) and M-MuLv reverse transcriptase.

The levels of IFITMs mRNA and intracellular HBV genomic DNA were determined by RT-qPCR analysis using SYBR Green Premix (Takara, Tokyo, Japan) on a LightCycler® 480II (Roche, Basel, Switzerland) system. The expression of the target genes was normalized to glyceraldehyde 3-phosphate dehydrogenase (GAPDH) by the ΔΔCt method. β-actin was used as control. Primers were as follows: IFITM1, forward: 5’-CCCCAAAGCCAGAAGATGCACAAGGAG-3’, reverse: 5’-CGTCGCCAACCATCTTCCTGTCCCTAG-3’; IFITM2, forward: 5’- CATCATCATCCCAGTGTTGG-3’, reverse: 5’-GATAAAGGGCTGATGCAGGA-3’; IFITM3, forward: 5’-CAAGGAGGAGCACGAGG-3’, reverse: 5’-TTGAACAGGGACCAGACG-3’; β-actin, forward: 5’-CTCTTCCAGCCTTCCTTCCT-3’, reverse: 5’-AGCACTGTGTTGGCGTACAG-3’; GAPDH, forward: 5’-GATGGCAAGATCTTCTGCGTG-3’, reverse: 5’-CCGTCGACTCACAGGAAATAGTCGGC-3’.

### 2.8. Small Interfering RNA (siRNA)-Mediated Knockdown

The siRNAs were designed using Oligo 6.0 software, and synthesized by GenScript Co. Ltd. (Nanjing, China) as follows: siIFITM1: siRNA1: 5’-AAACCUUCACUCAACACUUCCUU-3’, siRNA2: 5’-AAACUUAAGAGAAAUACACACUU-3’; siHBc: siRNA1: 5’-AAACUUUACUGGGCUUUAUUCUU-3’, siRNA2: 5’-AAGAGAAACUGUCCUUGAGUAUU-3’; siControl: 5’-GUAUAUAAGCAAGCAUUACUU-3’. All the siRNAs were transfected using RNAiMAX (Invitrogen) according to the manufacturer’s instructions.

### 2.9. Cell Viability

One hundred microliters of HepG2 or HepG2.2.15 cells of the same passage number were seeded into 96-well plates at a density of 2 × 10^3^ cells per well. After culturing for 24 h, cells were transfected with pGC-FU-Flag vector, siRNA, or pGC -FU-Flag-BAF200 and incubated for 24 h, then treated with 0, 200, 400, 600, 800, or 1000 U/mL IFNα for 24 h. Afterwards, cell viability was assessed using the MTT assay. The medium was refreshed and 5 mg/mL 3-(4,5-dimethlthiazol-2-yl)-2,5-diphenyltetrazolium bromide (MTT, Sigma-Aldrich) was added at 20 μL per well. After incubating for 2 h, the medium was removed, and cells were lysed in 100 μL DMSO. Finally, absorbance was measured by a microplate reader (Thermo Scientific) at 490 nm.

To ensure the results of the MTT assays, a trypan blue exclusion assay was performed. After the transfection and treatment of IFNα, cells were harvested. Then trypan blue (Sigma-Aldrich) was added to the cell suspension to a final concentration of 0.04% (*w*/*v*), and the mixture incubated at room temperature for 5 min. Ten microliters of the suspension was transferred to a hemocytometer and viable cells were counted. The test was repeated at least three times.

### 2.10. Enzyme Linked Immunosorbent (ELISA) Assay

HepG2 cells were transfected with pHBV1.31 and pGC-FU-Flag-BAF200, or together with pCMV-HBc-HA, in a 1:1 ratio. After treatment with IFNα for 72 h, the supernatant was collected to detect the levels of the hepatitis B surface antigen (HBsAg) and hepatitis B E antigen (HBeAg) using a commercial ELISA kit (Neobioscience, Hangzhou, China) and the HBV DNA copy number was quantified by real-time PCR with a commercial PCR-Fluorescence Quantification Kit (Bioer, Hangzhou, China).

### 2.11. HBV DNA Analysis

For the extraction of the nucleic acid, HepG2 cells were collected at 96 h post-transfection and lysed in a precooling lysis buffer (0.5% NP-40, 50 mM Tris-HCl [pH 7.0]). After centrifugation at 10,000× *g* for 1 min, the nuclei were pelleted, and the supernatant was adjusted with 10 mM MgCl_2_ and treated with DNase I for 1 h at 37 °C to remove the free DNA. The mixture was incubated at 75 °C for 15 min in the presence of 10 mM EDTA to inactivate the enzymes, then cultured with proteinase K in the presence of 1% SDS to digest proteins. At last, the nucleic acids were purified by phenol-chloroform extraction and ethanol precipitation.

For the extraction of extracellular encapsidated HBV DNA, free DNA and enzymes of 10 μL cell culture supernatant were removed according to the methods mentioned above. Then, the mixture was added to 100 μL of a lysis buffer (20 mM EDTA, 20 mM Tris-HCl, 0.5% SDS, and 50 mM NaCl) containing proteinase K and incubated at 50 °C overnight. After this, HBV DNA was isolated by phenol-chloroform extraction and ethanol precipitation. HBV DNA was subjected to real-time by PCR using primers (5’-AGAAACAACACATAGCGCCTCAT-3’ and 5’-TGCCCCATGCTGTAGATCTTG-3’) and probe (5’-TGTGGGTCACCATATTCTTGGG-3’).

### 2.12. Statistical Analysis

Data were presented as mean ± standard deviation (SD). All experiments were repeated three times. The statistical analysis was assessed using the Student’s *t*-test. Differences were considered statistically significant at *p* < 0.01 (**).

## 3. Results

### 3.1. HBc Interacts with BAF200

To dissect the function of HBc (Figure 1a), we screened a human fetal brain cDNA library for novel HBc-interacting proteins using a yeast two-hybrid system. Co-transformants were selected on SD/-Leu/-Trp and were validated by growth on SD/-Leu/-Trp/-Ade/-His/X-α-gal (Figure 1b). Then, positive colonies were sequenced. Finally, the C-terminal of BAF200 (BAF200C, aa 1419–1773) was identified as one of the strongest binding partners of HBc in AH109. BAF200C contains ZnF domains and can bind to DNA, RNA, or proteins [15] (Figure 1a). Because this interaction might play an important role in HBV-infected hepatocytes, our studies focused on the function of HBc interaction with BAF200.

To verify the two-hybrid results, co-IP assays were performed. First, BAF200C was co-transfected with either empty vectors or HBc into 293T cells, then the whole cell lysate was immunoprecipitated by an anti-Flag antibody and then subjected to western blot by anti-HA antibodies to detect the interacting proteins. The results indicate that the expressed C-terminal of BAF200 co-precipitated with HBc (Figure 1c). Then, HBc interaction with full-length BAF200 was further assessed in HepG2 cells. IP was performed against HA-tagged HBc and the co-precipitation was detected with the anti-Flag antibody. The data showed that overexpressed BAF200 co-precipitated with HBc (Figure 1d).

To investigate the endogenous interaction of BAF200 and HBc, HepG2-NTCP cells were infected with or without HBV by inoculation with or without the supernatants of HepaAD38 cells. Exogenous expression of sodium taurocholate co-transporting polypeptide (NTCP) in human HepG2 cells (HepG2-NTCP) rendered them susceptible to HBV/HDV infection [24]. HepG2-NTCP cells were infected with HBV by inoculation with or without the supernatants of HepaAD38 cells [23,24]. The lysates of HepG2-NTCP cells were immunoprecipitated by HBc antibodies or BAF200 antibodies, then subjected to western blot by BAF200 antibodies or HBc antibodies to detect the interacting proteins (Figure 1e). The result indicated that endogenous BAF200 also co-precipitated with endogenous HBc, as seen in overexpressed proteins. Collectively, the co-IP results confirm the binding between HBc and BAF200.

### 3.2. HBc Suppresses IFITM1 Expression

**Figure 2:**
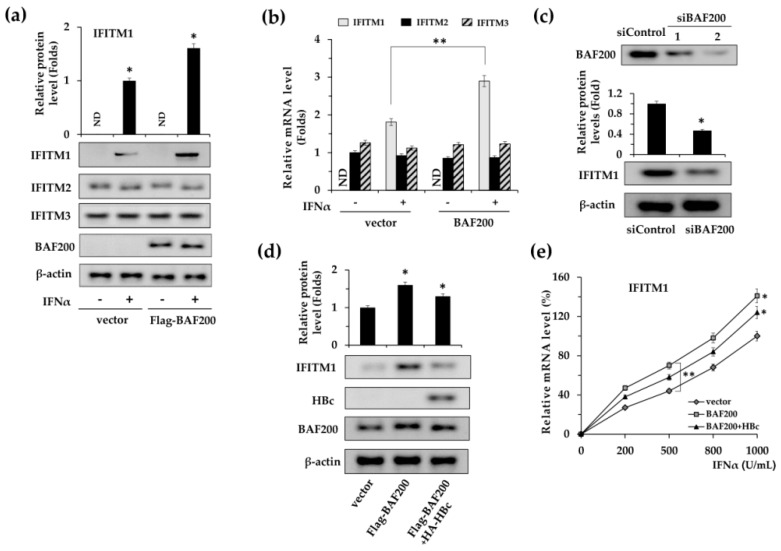
HBc regulates IFITM1 expression by binding to BAF200. (**a**) HepG2 cells were transfected with pGC-FU-Flag vector or pGC-FU-Flag-BAF200, treated with IFNα (500 U/mL) or not for 18 h, then IFITM 1, 2, and 3 expression levels were measured via western blot using antibodies against IFITM 1, 2, and 3 and normalized to the GAPDH mRNA level. Results are standardized to a value of 1 for IFITM2 mRNA level of empty vector transfection control without IFNα treatment. (**b**) Total RNA was extracted and the mRNA levels of IFITM 1, 2, and 3 were detected via RT-qPCR. (**c**) HepG2 cells were transfected with control siRNA (siControl) or siBAF200 and then treated with IFNα (500 U/mL) for 18 h. Levels of IFITM1 and β-actin proteins were determined by western blot using the corresponding antibodies. (**d**) The effect of HBc overexpression on IFITM1 expression levels was measured by western blot assays. (**e**) IFITM1 transcription levels under induction of IFNα at different concentrations were examined via RT-qPCR assays, and normalized to the GAPDH mRNA level. The vector transfection control treated with 1000 U/mL IFNα was designated as 100%. *, *p* < 0.05; **, *p* < 0.01.

Because BAF200 was reported to be a critical regulator of IFN signaling [13], we first focused on the effect of BAF200 on the expression of IFITMs, which are IFNα effectors. The impact of overexpressed BAF200 on IFITM1, 2, and 3 was examined via protein (Figure 2a) and mRNA (Figure 2b) levels. The results demonstrated that BAF200 up-regulated IFITM1 protein expression (Figure 2a) and significantly increased the mRNA expression (Figure 2b). However, no apparent effect of BAF200 was observed on the expression of IFITM2 and IFITM3 (Figure 2b). Furthermore, we observed that the IFITM1 expression level was suppressed 2.1-fold when endogenous BAF200 was silenced in HepG2 cells (Figure 2c). The data suggest that BAF200 specifically mediates basal level expression of IFITM1.

Next, the effect of the HBc interaction with BAF200 on IFITM1 expression was further investigated. We co-transfected HBc and BAF200 into HepG2 cells, treated the cells with 500 U/mL IFNα, and detected the expression of IFITM1 by western blot (Figure 2d). The results indicate that BAF200 enhanced IFITM1 expression, while the enhancement was partially reduced when HBc was co-transfected.

To further examine the effect of IFNα on HBc function, we treated the transfected cells with IFNα at various concentrations from 200 U/mL to 1000 U/mL for 24 h and examined the IFITM1 mRNA levels (Figure 2e). As expected, HBc impaired the enhancement of IFITM1 mRNA expression by BAF200. However, compared to the control, expression was inhibited to a similar degree upon IFNα stimulation with different concentrations.

Taken together, the data demonstrates that HBc can down-regulate IFITM1 expression via binding to BAF200, whereas the binding is irrelevant to the stimulation of IFNα.

### 3.3. HBc Downregulates IFITM1 Expression by Modulating PBAF Stability

**Figure 3:**
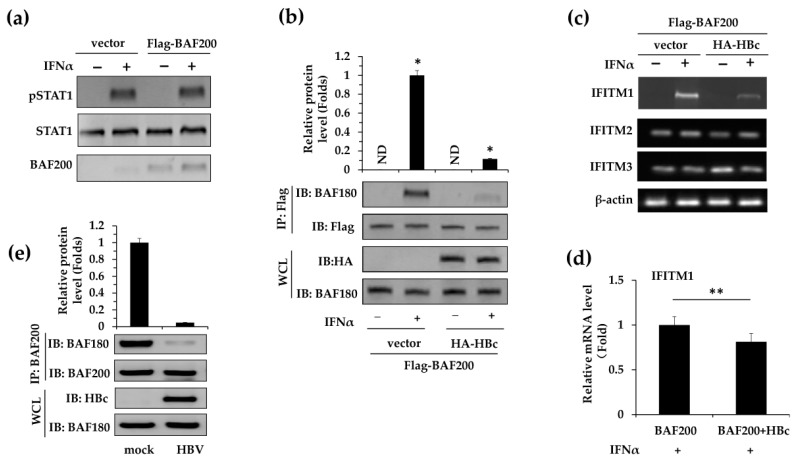
HBc downregulates IFITM1 expression by modulating PBAF stability. (**a**) The pCMV-Flag vector and pCMV-Flag-BAF200 were transfected into HepG2 cells, with or without IFNα (500 U/mL) treatment for 24 h. Phosphorylation of STAT1 was detected via western blot using antibodies against phosphorylated STAT1 (pSTAT1). Meanwhile, STAT1 and BAF200 were detected using anti-Flag and anti-STAT1 antibodies as control. (**b**) HepG2 cells were co-transfected with pGC-FU-Flag-BAF200 and pCMV-HA vector or pCMV-HA-HBc, with or without IFNα (500 U/mL) treatment for 24 h. Co-IP assays were performed with anti-Flag antibody, then BAF200–BAF180 immuno-complexes were detected by western blot assays using anti-BAF180 antibody or anti-Flag antibodies (control). Input control assays were performed in WCL using anti-HA or anti-BAF180 antibodies. Meanwhile, total mRNA was extracted to examine the effect of BAF200 and HBc combination on IFITM1, 2, 3 transcriptions by RT-PCR (**c**). β-actin was detected as control. IFITM1 transcription level was determined using RT-qPCR and normalized to the GAPDH mRNA level. (**d**) The Flag-BAF200 transfection with IFNα treatment was designated as 1. *, *p* < 0.05; **, *p* < 0.01. ND = Not Detected. (**e**) HepG2-NTCP cells were incubated with supernatants isolated from the supernatants of HepaAD38 cells cultures (containing HBV) at 1000 GEq for 16 h or not (mock). Co-IP assays were performed with BAF200 antibodies, and immuno-complexes were detected by western blot assays using BAF180 antibodies or BAF200 antibodies. Input control assays were performed in WCL using HBc antibody or BAF180 antibody.

Furthermore, the mechanism about how HBc regulated IFITM1 expression was explored. Since the JAK–STAT1 signaling pathway has been reported to be essential to IFNα-induced antiviral activities [26], we examined whether it was involved in BAF200-mediated IFITM1 expression. Since the phosphorylation of STAT1 is a necessary step for JAK–STAT1 signaling, we used western blot assays to detect phosphorylated STAT1 (pSTAT1) in HepG2 cells with or without BAF200 (Figure 3a). The results showed that pSTAT1 appeared only upon IFNα stimulation, independent of the presence of BAF200.

It has been shown that BAF200 is essential for the stability of PBAF, and the depletion of BAF200 leads to the complete inactivation of PBAF [13]. Hence, we predicted that the interaction of HBc with BAF200 would interrupt the stability of PBAF complexes, leading to potential BAF200-dependent IFITM1 expression. To verify this hypothesis, co-IP assays were carried out to analyze the effect of HB on BAF180-BAF200 immuno-complexes in HepG2 cells (Figure 3b). IP was performed against Flag-BAF200 and the co-precipitation of BAF180 was probed. It demonstrated that HBc co-transfection along with BAF200 profoundly reduced the co-precipitation of BAF180–BAF200 immuno-complexes when compared with the BAF200 transfected alone condition. Endogenous BAF180–BAF200 interaction was further assessed in HBV infected HepG2-NTCP cells (Figure 3e). The co-IP assay showed that the concentration of BAF180–BAF200 complexes was reduced more than 50-fold compared to the non-infected mock control. These results imply that HBc de-stabilizes the PBAF complex by preventing the BAF180–BAF200 interaction, probably by competitive binding to BAF200.

We next determined whether overexpression of HBc regulated IFITM1 transcription in vitro. Flag-BAF200 was transfected with or without HA-HBc into HepG2 cells. Cells were later treated with IFNα (500 U/mL) and total mRNA was extracted to detect transcription levels of IFITM1, 2, and 3 by RT-qPCR. The data indicates that, upon IFNα stimulation, the suppression of IFITM1 expression by HBc is specific (Figure 3c) and statistically significant (Figure 3d) in vitro. Collectively, the data demonstrates that HBc interacts with BAF200, and the HBc–BAF200 interaction prevents the BAF180–BAF200 interactions that might abolish PBAF complex stability, which in turn regulates the suppression of IFITM1 transcription.

### 3.4. HBc Partially Restores IFN-α Inhibition of HBV Expression in Transfected Hepatoma Cells

**Figure 4:**
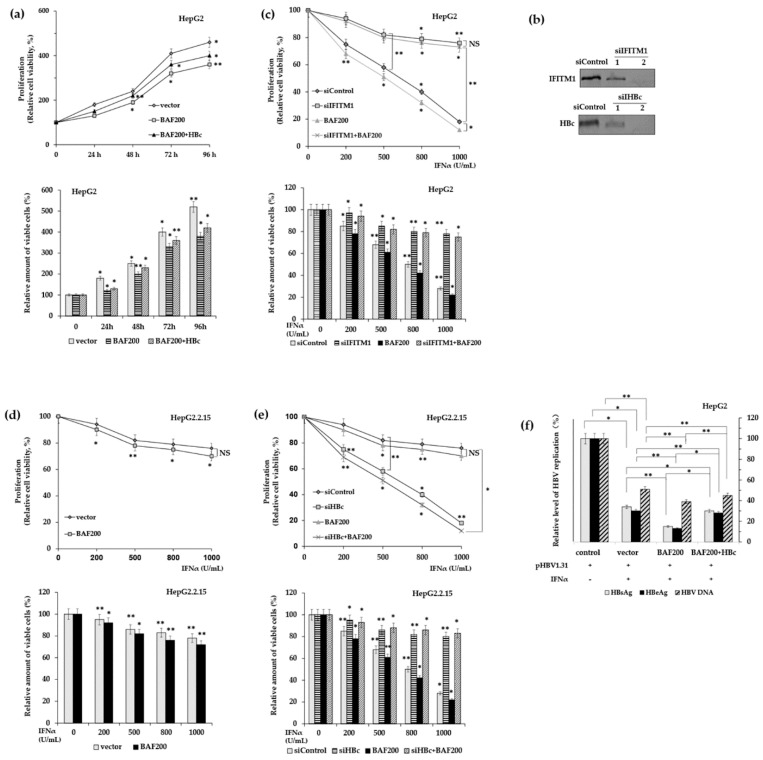
The impacts of HBc on IFNα anti-virus activity in vitro. (**a**) HepG2 cells were transfected with BAF200 with or without HBc and stimulated with 500 U/mL IFNα for indicated time. (**b**) Deletion efficiency of siRNA-oligos were examined in HepG2 cells using western blot. pGC-FU-Flag vectors, siRNA, or pGC-FU-Flag-BAF200 pasmids were transfected into HepG2 cells (**c**) or HepG2.2.15 cells (**d**,**e**), then treated with IFNα in indicated concentration for 24 h. Afterwards, the cell viability was measured using MTT assay and direct cell counting via trypan blue exclusion assay (**a**,**c**–**e**). (**f**) HepG2 cells were transfected with pHBV1.31 and pGC-FU-Flag-BAF200, or co-transfected with pCMV-HA-HBc, then treated with 500 U/mL IFNα for 72 h. HBV replication levels were determined in the supernatants by measuring HBsAg and HBeAg concentration using ELISA and HBV DNA copy numbers using RT-qPCR. The empty vector and pHBV1.31 were co-transfected in the non-IFNα induced condition as control. The control without IFNα treatment was designed as 100% (**a**,**c**–**f**). *, *p* < 0.05, **, *p* < 0.01, NS = non-significant.

Finally, we evaluated the effect of HBc on the antiviral activities of IFTIM1 in HBV-infected cells. Firstly, the impact of HBc on the anti-proliferation action of IFITM1 was investigated via siRNA-mediated knockdown in HepG2 cells and HepG2.2.15 cells. Proliferation of indicated cells was assessed by MTT assays and viable cell counting. Indeed, in HepG2 cells and HepG2.2.15 cells, the data from direct cell counting were consistent with those obtained from MTT assays (Figure 4a,c,d,e). In HepG2 cells, the cell viability increased with the time of IFNα stimulation (Figure 4a) and decreased with the concentration of IFNα (Figure 4c). Unter treatment of 500 U/mL IFNα, BAF200 enhanced the inhibitory effect of IFNα, and overexpression of HBc partially recovered the cell viability (Figure 4a), however, the siRNA mediated knock down of IFTIM1 increased the cell viability robustly, about two–four-fold compared to the control (Figure 4c). The results suggest that IFITM1 makes major contributions to IFNα inhibitory activities for HBV replication. Next, we focused on the role of HB. In HepG2.2.15 cells, which contain a stably integrated HBV genome and produce endogenous HBc, overexpression of BAF200 promoted the inhibition of IFNα (Figure 4d), however when HBc was silenced by siHBc, cell viability was reduced substantially (Figure 4e). Consequently, the results demonstrate that HBc antagonizes the suppression of IFTIM1 and the proliferation of HBV-infected cells in vitro.

Next, the HBc effects on the inhibition of IFITM1 on HBV replication were measured. In Figure 4e, pHBV1.31, which contains 1.3-fold HBV genome and can produce endogenous HBc, was co-transfected with empty vector Flag-BAF200 or HA-HBc into HepG2 cells. After treating the cells with 500 U/mL IFNα for 72 h, supernatants were collected to determine the HBV replication level by detecting the quantitation of HBsAg, HBeAg, and HBV DNA copy numbers (Figure 4f). In the control, empty vector and pHBV1.31 were co-transfected, and it was observed that IFNα stimulation inhibited the HBV replication level significantly. In contrast to the control, when BAF200 was overexpressed, the concentration of HBsAg and HBeAg in the supernatant was reduced about one-fold, and HBV DNA copies were also suppressed up to five-fold. As expected, the IFNα inhibition was restored when HBc was co-transfected with BAF200. Notably, HBsAg and HBeAg levels increased to similar levels as the control, with a slight elevation in the HBV DNA copy numbers.

Collectively, the results illustrate that HBV evades IFNα mediated antiviral activity in vitro by downregulating the expression of IFNα effector. However, overexpressed HBc had little observed impact on the recovery of HBV DNA levels, implying that IFITM1 is not the only primary IFNα-inducible ISG that suppresses HBV DNA replication.

## 4. Discussion

HBV replication is not directly cytotoxic to cells, while the host immune responses in infected hepatocytes are the main determinants of hepatocellular injury and HBV pathogenesis. Innate immunity is important in controlling viral spread immediately after infection and initiates efficient development of an adaptive immune response. The early phase of a viral infection is mainly characterized by the production of cytokines, type I IFN, and natural killer (NK) cells [27]. Type I IFNs can be triggered directly by virus replication through the detection of viral RNA or DNA, and play a critical role in the immune recognition of HBV. They can be triggered directly by viral replication via detecting the presence of viral RNA or DNA and induce a large number of effectors working in combination to achieve a fully functional antiviral state [4,28].

IFN effectors target different steps of the viral life cycle, limiting the propagation and spread of the virus and restricting viral infection. Therefore, interferon therapy remains one of the therapeutic strategies for HBV infection. In fact, the majority of CHB patients treated with IFNα cannot acquire a long-lasting sustained response, especially those with a high viral load. Data from animal models have shown that IFN effectors were rapidly induced upon infection with HBV [28]. However, the HBV-induced IFN responses are weak [29], and that is why acute HBV infections usually show a lack of clinical symptoms. Studies have shown that the clinical outcomes in CHB patients are primarily determined by the interaction between HBV replication and host immune responses [30]. As an important effector of Type I IFNs, MxA exhibits strong anti-viral activity against HBV, however, HBV down-regulates MxA expression significantly via the interactions between HBc and the interferon-inducible MxA promoter [31,32]. In addition, reports have indicated that under the stimulation of IFNα, the IFN-signaling pathway is generally blocked, however the formation of STAT1 and ISGF3 were evenly enhanced in HepG2.2.15 cells [33]. Here, our study shows that HBc inhibits the IFNα-induced IFITM1 transcription through disturbing the stability of PBAF complex in vitro, independent of the JAK–STAT1 pathway (Figure 4a). Moreover, HBc exhibits anti-apoptosis activity by inhibiting TRAIL-induced expression of the pathway-related death receptor in hepatocytes [34]. HBc acts as a tumor suppressor as well, by blocking the transcription of the human tumorigenesis-associated genes, IFNβ and p53 [11,35].

**Figure 5:**
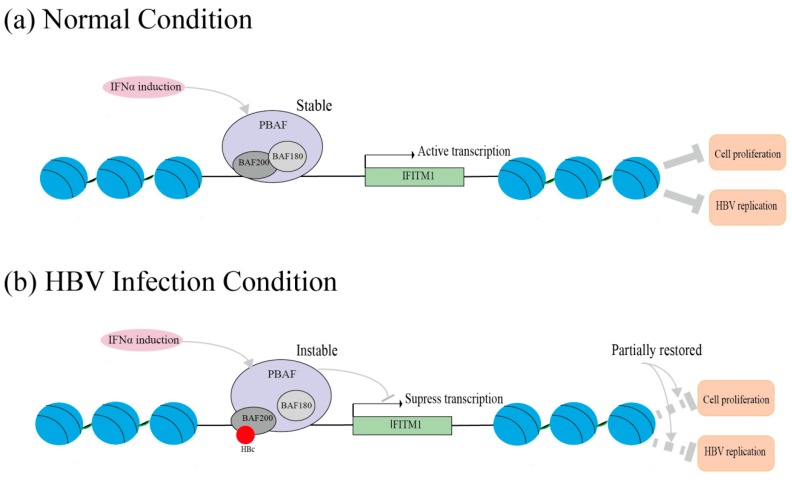
Schematic diagram of the mechanism for HBc downregulation of IFTM1 expression. The diagram illustrates the HBc function under normal and HBV infection conditions. (**a**) Under normal conditions, IFNα induction activates PBAF complexes, leading to the exposure of interferon-stimulated response elements (ISRE) and the initiation of IFITM1 transcription. (**b**) Under HBV-infected condition, the interaction of HBc with BAF200 disturbs the stability of PBAF complex, resulting in inefficient process of PBAF complex and the suppression of IFITM1 transcription.

Notably, our data indicates that the inhibitory effect of IFITM1 on HBV DNA replication seems weak. A potential explanation is HBV integration into the host genome. The HBV genome can present as non-integrated cccDNA, which serves as a template for replication and can persist even after HBsAg loss [36,37]. HBV manages to escape immunological recognition in early infection, remaining undetected and spreading for nearly five weeks [38]. The use of cccDNA as a transcriptional template in the nucleus likely contributes to HBV’s capacity to limit detection in hepatocytes [37]. In addition, HBV evades the host response through a complex combination of processes that include signaling interference, effector modulation, and continual viral genetic variation [37]. Reports have shown that HBV polymerase and HBx protein directly inhibit the cellular machinery that detects replication intermediates [29,33]. Here, our study provides novel evidence that HBV counteracts IFNα antiviral activities through effector modulation, i.e., the downregulation of IFITM1 expression by HBc. The mechanism of the downregulation is illustrated as follows. Under normal conditions, IFNα induction activates PBAF complexes, which consist of BAF200, BAF180, and other subunits. The activation of the PBAF complex triggers histone sliding on chromatin DNA, leading to the exposure of interferon-stimulated response elements (ISRE). In this way, PBAF complexes facilitate the initiation of IFITM1 transcription (Figure 5a). Actually, BAF200 is essential for the stability of the PBAF complex. Under HBV-infected condition, the interaction of HBc with BAF200 competes against the binding between BAF180 and BAF200, resulting in the loss in PBAF complex stability. Thus, the efficient process of the PBAF complex is interrupted, leading to the suppression of IFITM1 transcription (Figure 5b). It provides details for the mechanism of HBV escape from IFNα-induced immune elimination. Moreover, our future work will continue to explore the intrahepatic effect of HBc on HBV replication using HBV-transgenic mice.

Taken together, our data demonstrate that HBc downregulates IFITM1 expression through interactions with BAF200, rather than the JAK–STAT1 pathway. The results also show that HBc partially restores IFITM1 antiviral effects on cell proliferation and HBV replication in infected cells in vitro by disturbing the stability of PBAF. It enriches the mechanism that HBV counteracts the innate immunity of IFNα, revealing a potential target for novel therapeutic strategies of HBV infection.
